# Educating Health Professionals about Drug and Device Promotion: Advocates' Recommendations

**DOI:** 10.1371/journal.pmed.0030451

**Published:** 2006-11-07

**Authors:** Peter R Mansfield, Joel Lexchin, Leana S Wen, Luisella Grandori, Christopher P McCoy, Jerome R Hoffman, Joana Ramos, Jon N Jureidini

## Abstract

Mansfield and colleagues outline the recommendations from four advocacy groups for improving the education of health professionals on promotion of drugs and devices.

This Health in Action provides recommendations for improving education for health professionals about pharmaceutical and device promotion, which includes any activity that can increase sales of pharmaceuticals or devices. The recommendations were produced by an iterative E-mail discussion among representatives of four organizations: the American Medical Student Association, Healthy Skepticism Inc., No Free Lunch, and PharmAware (Box 1).

We hope these recommendations will inform, stimulate, and support educators of health professionals to develop improved education about pharmaceutical and device promotion. We will survey educators to seek their views on these recommendations.

## Background

In the promotion of rofecoxib (Vioxx), “drug marketing got well ahead of the science” [1]. The successful hormone-replacement-therapy marketing campaign “convinced physicians that so called HRT [hormone-replacement therapy] prevented cardiovascular disease before one single clinical trial with cardiovascular disease end points had ever been done” [2]. These are just two examples of how misleading promotion can be a major threat to health [1,2].

There were an estimated 88,000–140,000 excess cases of serious coronary artery disease attributable to rofecoxib in the United States alone [3]. The number of women harmed by severe adverse effects of hormone-replacement therapy, including breast cancer, may have been even larger because hormone-replacement therapy was used for longer, but we are not aware of any reliable estimate. Reforms are needed to reduce the risk of similar events occurring again [4].

The US Accreditation Council for Continuing Medical Education states that “residents must learn how promotional activities can influence judgment in prescribing decisions and research activities through specific instructional activities” [5]. World Health Assembly resolution 52.19 urges member states to “integrate the rational use of drugs and information on commercial marketing strategies into training for health practitioners at all levels.” However, a recent worldwide survey of education about pharmaceutical promotion in medical and pharmacy schools found that “in most cases … students devoted one half day or less to this topic during their professional training; in nearly one third of cases, medical faculties devoted only 1–2 hours” [6]. That survey also found wide variations in objectives, ranging from aiming to “increase students' ability to extract beneficial information from drug promotion” to aiming to “increase students' use of independent information sources.”

**Box 1. The Four Organizations Calling for Action**

**American Medical Student Association**

(http://www.amsa.org)

An association of nearly 60,000 doctors-in-training with aims including: improving health care and healthcare delivery to all people and promoting active improvement in medical education.

**Healthy Skepticism Inc.**

(http://www.healthyskepticism.org)

An international organization aiming to improve health by reducing harm from misleading pharmaceutical promotion.

**No Free Lunch**

(http://www.nofreelunch.org, http://www.nofreelunch.uk, http://www.nograziepagoio.it)

An international network aiming to improve patient care by encouraging health care providers to practice medicine on the basis of scientific evidence rather than on the basis of pharmaceutical promotion.

**PharmAware**

(http://www.pharmaware.co.uk)

A group of British medical students who aim to change doctors' relationships with the pharmaceutical industry.

## Recommendations

The American Medical Student Association, Healthy Skepticism. Inc., No Free Lunch, and PharmAware recommend four objectives for education about pharmaceutical and device promotion (Box 2). We recommend that all four objectives should be pursued throughout all health professionals' careers: during every year of initial professional education, specialist training, and continuing professional education. Education should use methods that are effective for changing behavior, including interactive experiences and involvement of opinion leaders [7]. Education for health professionals should never, we believe, be funded by companies promoting drugs or devices [8–12].

Our recommendations are based mostly on studies of psychology students' responses to persuasion, and medical students' and physicians' responses to pharmaceutical promotion. There is little relevant published evidence on the effects of promotion on other kinds of health professionals or on the promotion of devices. These research gaps deserve priority. However, the available evidence [13–16] leads us to believe that our recommendations are appropriate for all health professionals and are relevant to promotion of all therapeutic and diagnostic devices.

Our recommended objectives challenge widely held beliefs. Consequently, we believe that educators should assess and address students' and professionals' initial beliefs about drug and device promotion, so as to maximize progress toward the beliefs required for appropriate use of drugs and devices reflected in our recommendations below.

**All health professionals should be educated explicitly about decision making and evaluation of evidence and promotion.** Education for health professionals should aim to improve the quality of decision making. This includes studying the areas of psychology, logic, economics, ethics, and statistics that are relevant to making good decisions, evaluating evidence, and evaluating the promotion of drugs and devices. For example, insights from all those disciplines are integrated in an interactive educational Website module produced by Healthy Skepticism Inc., for the Royal Australasian College of Physicians (http://www.racp.edu.au). This Web site introduces the topics of human decision making and vulnerability to persuasion [17–21], pharmacoeconomics [22], ethical problems arising from gift taking [11], and common misunderstandings of statistics [23–25].

**Box 2. Four Objectives for Education about Pharmaceutical and Device Promotion**

All health professionals should be aided in the following ways:

- Educated explicitly about decision making and evaluation of evidence and promotion.
- Helped to understand that there is no proven method for enabling them to gain more benefit than harm from promotion.
- Helped to understand their responsibility to avoid pharmaceutical and device promotion.
- Educated explicitly about the most reliable sources of information.

**Health professionals should be helped to understand that there is no proven method for enabling them to gain more benefit than harm from promotion.** There is strong evidence that exposure to pharmaceutical promotion correlates with medically inappropriate and wasteful use of pharmaceuticals [26]. However, there is a wide range of opinions among health professionals about the benefit-to-harm ratio of promotion, and their own susceptibility to it. Many believe they are capable of distinguishing between justified and unjustified promotional messages. However, few health professionals have much knowledge of misleading promotional techniques, and such knowledge does not reliably protect people from being misled [18,27].

Resistance to misleading promotion can be increased somewhat by helping people move from overconfidence in their abilities to understanding that they are vulnerable. [27–29]. Consequently, education for health professionals should explain that while knowledge of misleading promotional techniques may help them avoid being misled sometimes, there is no proven method for enabling them to gain more benefit than harm from exposure to pharmaceutical promotion. People who are confident in their ability to distinguish justified from unjustified promotional messages may be operating under an illusion, as many influence techniques are very difficult for humans to evaluate and there are no proven methods for sorting them.

One method for reducing such overconfidence is to first expose participants to a single example of misleading drug or device promotion, allow them to express unjustified beliefs, debunk those beliefs, and then finally explain the misleading techniques that were used. This method enables participants to understand that they are personally vulnerable to being misled by promotion [18,27]. The aim of this method is to decrease confidence as opposed to the common educational objective of increasing confidence.

One example of such a strategy for reducing confidence is an educational session held at the University of California, Los Angeles, in which a university pharmacist was introduced to medical students as “a pharmaceutical sales representative.” The “representative” proceeded to mimic standard sales techniques with regard to a given drug, while offering the students a “free” breakfast. The “representative” was then asked to leave the room for 20 minutes, to allow students to discuss the presentation. The “representative” then returned to reveal herself as a university pharmacist, and to explain and critique the techniques that had been used. This session was shown to be effective in reducing students' overconfidence in their “skills” at critical appraisal of sales representatives [29].

**Health professionals should be helped to understand their responsibility to avoid pharmaceutical and device promotion.** Because all health professionals are vulnerable to being misled, we believe that they have a professional, ethical, and fiduciary responsibility to patients to take all practical steps to avoid drug and device promotion [30–32]. This responsibility includes a personal policy of refusing to accept personal gifts and one-toone visits from sales representatives, and supporting organizational policies against such practices [33]. Meetings of groups of health professionals with sales representatives may be less harmful than one-to-one meetings, but it is unlikely that this type of activity will be found to do more good than harm compared with no such meetings. Education for health professionals should not include exposure to pharmaceutical or device promotion [8,30], except for educational examples that are immediately debunked (as discussed above).

There is observational evidence of benefits from limiting contact between pharmaceutical company sales representatives and doctors in training. In 1992, McMaster University implemented a policy that restricted interactions between internal medicine residents and sales representatives during daytime hospital activities. This policy included bans on sales representatives attending educational meetings and a ban on drug company–funded lunches. Three years after training under that policy, internists were more skeptical of, and had less contact with, sales representatives than internists trained before that policy or trained at the nearby University of Toronto, where there was no policy [34]. Other observational studies have found that doctors who are more skeptical of sales representatives and/or have less-frequent contact with them tend to be more judicious prescribers. [35–39]

Education about the professional responsibility to avoid promotion should be integrated with hands-on education about how to interact with others who have different views. This is addressed by the American Medical Student Association's PharmFree Campaign stepwise module that begins in the first year of medical school and continues through residency training. This module promotes the vision that “all medical students will learn about the ethics of drug company interaction with health professionals and make the rational, informed decision to eschew “free” gifts from the pharmaceutical industry throughout the training career” (http://www.amsa.org/prof/pharmfree.cfm).

**Health professionals should be educated explicitly about the most reliable sources of information.** Health professionals should receive explicit education about the availability, strengths and weaknesses of the least-biased, clinically useful sources of information, and the need to keep themselves up to date with the best information sources available. This should include use of such information as part of routine patient care by themselves and by role models. Professional associations and governments should actively develop programs to ensure that these sources are readily available to health professionals. Health professionals should be educated on how to convey reliable information to other health professionals and to the public, so as to provide a superior alternative to information from pharmaceutical and device companies.

## Conclusion

Our recommendations are necessary but not sufficient for removing the adverse influence of promotion on health professionals. Improved regulation and redesigned incentive systems are also needed [4,40]. Our recommendations challenge deeply held beliefs, so implementation will be difficult. However, pharmaceutical and device promotion causes much more harm than is generally realized [26], so significant reforms deserve priority. Our hypothesis—that implementing our recommendations will lead to improved health-care outcomes and earn increased public trust in the ability of health professionals to provide optimal treatment—deserves to be tested.
